# Assessing Mitochondrial Energetics of Skeletal Muscle in the Study of Muscle Mobility and Aging (SOMMA)

**DOI:** 10.1093/geroni/igab046.484

**Published:** 2021-12-17

**Authors:** Bret Goodpaster, Peggy Cawthon, Stephen Kritchevsky, Anne Newman, Russell Hepple, Steve Cummings, Paul Coen

**Affiliations:** 1 AdventHealth, Orlando, Florida, United States; 2 California Pacific Medical Center, San Francisco, California, United States; 3 Wake Forest School of Medicine, Winston Salem, North Carolina, United States; 4 University of Pittsburgh, Pittsburgh, Pennsylvania, United States; 5 University of Florida, Gainsville, Florida, United States

## Abstract

Mitochondria produce energy as ATP that essential for muscle contraction and movement. We hypothesize that age-related decreases in the capacity to generate ATP in muscle plays a major role in loss of mobility with aging. In SOMMA, we use high-resolution respirometry to measure the activity of electron transport system (ETS) in permeabilized muscle fibers from muscle biopsies. This allows us to assay ETS function in a highly controlled ex vivo experiment at the myocellular level, removed from other potentially limiting physiological factors including supplies of substrates and oxygen. We are also measuring the maximal capacity to generate ATP (ATPmax) in vivo by 31PMRS. ATPmax reflects the rate of phosphocreatine replenishment via oxidative phosphorylation. Analysis from the first 113 participants indicates that ATPmax correlates with Maximal OXPHOS (r=0.27, P=0.005), and Maximal ETS capacity (r=0.17, P=0.08). This suggests that these approaches provide complementary information on skeletal muscle energetics.

